# Supplementary material for: A Novel Signature of CCNF-Associated E3 Ligases Collaborate and Counter Each Other in Breast Cancer
Source: Cancers (Basel). 2021 Jun 8;13(12):2873. doi: 10.3390/cancers13122873 (PMC8228695; doi:10.3390/cancers13122873)
Supplement: Supplementary file 1 [file cancers-13-02873-s001.zip › suppl+ WB/revised WB.pdf]

Figure 1. Original Images of Figure 5D showing full Western blots.

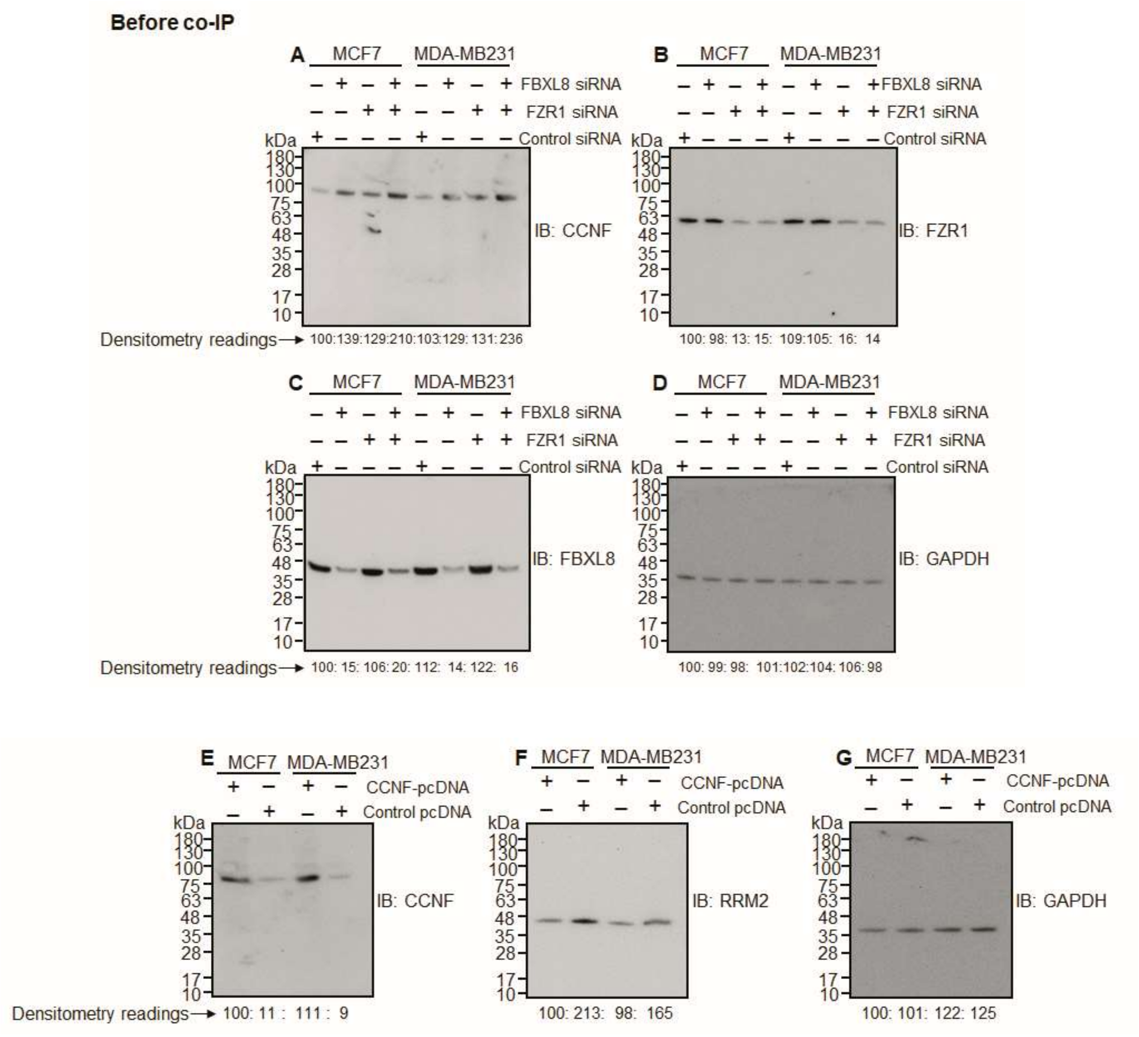

Figure 1. Original Images of Figure 5D for Blots. The original images of full blots for Figure 5D, showing Western blot detection of (A) CCNF, (B) FZR1, (C) FBXL8, (D) GAPDH, (E) CCNF, (F) RRM2 and (G) GAPDH. The densitometric scan are in absorbance unit (A.U.), shown at the bottom of each blot.

Figure 2. Original Images of Figure 5E showing full Western blots.

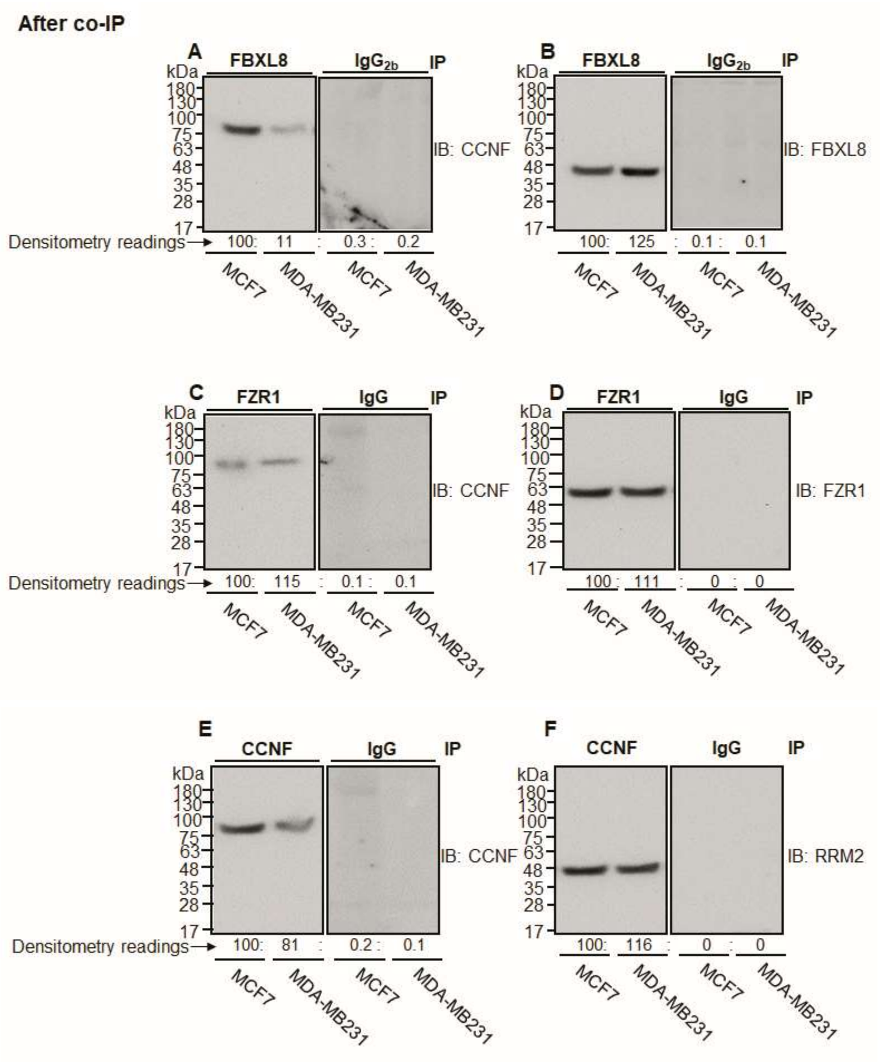

Figure 2. Original Images of Figure 5E for Blots. The original images of full blots for Figure 5E, showing Western blot detection of (A) CCNF, (B) FBXL8, (C) CCNF, (D) FZR1, (E) CCNF and (F) RRM2. The densitometric scan are in absorbance unit (A.U.), shown at the bottom of each blot.
