# Supplementary material for: A Novel Signature of CCNF-Associated E3 Ligases Collaborate and Counter Each Other in Breast Cancer
Source: Cancers (Basel). 2021 Jun 8;13(12):2873. doi: 10.3390/cancers13122873 (PMC8228695; doi:10.3390/cancers13122873)
Supplement: Supplementary file 1 [file cancers-13-02873-s001.zip › suppl+ WB/Supplementary Information.pdf]

**Supplementary Information**

**Materials and Methods**

*Tissue samples*

The primary samples used in this study were prepared as described previously [1]. In total, n=70 (35 tumor tissues and 35 normal tissues) from breast cancer patients were acquired from the BioBank, Taipei Medical University, Taiwan, following confirmation of the clinical diagnosis by biopsy and histological evaluation. All samples were collected with informed consent from the patients. The study was approved by TMU-JIRB (Taipei Medical University-Joint Institutional Review Board; IRB: N201803107). For paired normal tissue control, adjacent non-cancerous breast tissues were obtained from at least 2 cm away from the tumor node. *Ex vivo* experiments included: (i) NGS-based RNA-seq analysis, where five-pairs of the primary tissues (n=10) are used, including: 1-paired stage I patient, 3-paired stage II patients and 1-paired stage III patients (the corresponding clinicopathological information are shown in Supplementary Table S1); and (ii) immunofluorescence staining, where 30 pairs of the tissues (n=60) were used, including: 9-paired stage I patients, 11-paired stage II patients and 10-paired stage III patients (the corresponding clinicopathological information are shown in Supplementary Table S2). The H&E sections were obtained from the BioBank, Taipei Medical University, Taiwan. For immunofluorescence staining, paraffin-embedded tissues were sectioned at 5 μm thickness, dehydrated and blocked

in Ultravision Protein Block (Thermo Scientific TA060PBQ) for 10 min at room temperature. The sections were then incubated with primary and secondary antibodies overnight and 1 h, respectively. The stained tissue sections were observed under an Olympus microscope (Tokyo, Japan) and images were acquired using EOS Utility software (Canon).

*NGS (next-generation sequencing)-based RNA-Seq (sequence) analysis*

The primary samples used in this analysis were prepared as described previously [1]. The primary BRCA tissue samples were prepared as follows:

*(1) RNA isolation for NGS-based RNA-Seq.* Total RNA was extracted by Trizol® Reagent (Invitrogen, USA) following the manufacturer's instruction. Purified RNA was quantified at $OD_{260nm}$ by using Qubit (Life Technologies, Qubit® 2.0 Fluorometer, USA) and analysed using a bioanalyzer (Agilent, Bioanalyzer 2100 system, USA) with RNA 6000 labchip kit (Agilent Technologies, USA).

*(2) mRNA library preparation & NGS-based RNA-Seq.* All procedures were carried out according to the Illumina protocol, using Agilent's SureSelect Strand Specific RNA Library Preparation Kit (100SE, Single-End) for sequencing on Solexa platform. The sequences were determined using sequencing-by-synthesis technology via the TruSeq SBS Kit. Raw sequences were obtained from the Illumina Pipeline software

bcl2fastq v2.0, which was expected to generate 5M (million reads) per sample.

*(3) RNA-Sequence analysis.* The quality control of raw sequencing data was performed with FastQC (cite). Adapter and quality trimming were performed using cutadapt (phred cutoff: 20; minimum length: 25) (cite). Reads were mapped to the human reference genome using STAR aligner indexed with GRCh37 assembly and GENCODE Release 26 v26lift37 annotation. Gene expression was quantified using RSEM. The expected counts were used as input for the differential gene expression analysis between tumor and adjacent-normal samples using edgeR in the R environment. A false discovery rate (FDR) <0.05 was regarded as threshold to define genes that showed statistically significant differential expression. A log2 fold change (logFC) < 0 indicates under-expression, while logFC > 0 represents over-expression in the tumor samples.

*Quantitative real-time PCR (qRT-PCR)*

Samples were prepared as described previously [1]. Briefly, 24 h after treatment of cell lines, total RNA was extracted with TRIzol reagent (Invitrogen) and the RNA was reverse transcribed using random hexamers and HiSenScript™ RH(-) RT-PCR PreMix Kit (iNtRON Biotechnology, Seongnam, South Korea), following the manufacturer's instructions. The synthesized cDNA was used for real-time PCR

analysis using SYBR Green (Life Technologies) on a QuantStudio 3 Real-Time PCR System (Applied Biosystems, Foster City, CA, USA).

*Cell viability assay*

MTT {3-(4,5-dimethylathiazol-2-yl)-2,5-diphenyl tetrazolium bromide} and CTB (CellTiter Blue) obtained from Life Technologies (Carlsbad, CA, USA) and Promega (Madison, WA, USA), respectively, were used to determine the cell viability, according to the manufacturers' instructions. To measure the BRCA cell survival in response to presence or absence of FBXO43 (or FBXO15 or CCNF), both MCF7 and MDA-MB231 cells were treated with either (1) specific siRNA (targeting to FBXO43, FBXO15 or CCNF), control siRNA or PBS, or (2) CCNF-pcDNA3.1, control pcDNA3.1 or PBS for 16 h. The cells were then incubated with either 10 μl MTT reagent in 100 μl culture medium at 37°C for 4 h, or 10 μl CTB reagent at 37°C for 4 h. The metabolic activity of viable cells was measured at 570 nm using a Synergy H4 microplate reader (BioTek, Winooski, VT, USA) at time points indicated. Samples from each time point were normalized with corresponding PBS controls.

*Cell proliferation assay*

Cells were transfected with target-specific siRNA or control scrambled siRNA, or

CCNF-pcDNA3.1 or control pcDNA3.1 vector alone for 16 h. Then, Alamar blue was used to measure cell growth. The metabolically active cells were then determined at 570 nm using a Synergy H4 microplate reader (BioTek, Winooski, VT, USA) at time points indicated. For Trypan Blue dye exclusion, to assess total cell number, cells were scraped and resuspended in equal volumes of culture medium and trypan blue dye (0.4% solution; Gibco, Waltham, MA, USA) and counted using an improved Neubauer hemocytometer chamber.

*Cellular apoptosis assay*

CCNF-pcDNA3.1 plasmid construct was introduced transfected into MCF7 and MDA-MB231 cells; the control was empty vector and background control was PBS. After 24 h, the cells were examined for apoptosis. Early apoptosis was measured using Annexin V (BioLegend, San Diego, CA, USA) in conjunction with 7-AAD (BioLegend). Annexin V identifies surface-exposed phosphatidylserine, and 7-AAD is retained in late apoptotic cells. The Annexin $V^{+}$/7-$AAD^{-}$ cells were then analyzed using FACScan flow cytometer (FACSVerse, BD Biosciences, Franklin Lakes, NJ, USA) for a minimum of 10,000 events.

*Caspase-9 and -3 assays*

The approach was described previously cite. 24 h after treatment of CCNF-pcDNA3.1, control empty vector, or PBS control, apoptosis of MCF7 and MDA-MB231 cells were determined by examining the caspase-specific cleavages of activated caspase-9 and -3 (Abcam). The p-nitroanilide (p-NA) light emission was measured at 405 nm using a Synergy H4 microplate reader (BioTek).

*Cell migration and invasion assays*

Cell migration assay was carried out 24 h after transfection of the BRCA cells with CCNF-pcDNA3.1 or controls (empty pcDNA3.1 vector and PBS). Briefly, the cells were rinsed with PBS, treated with 0.05 % Trypsin-EDTA (Invitrogen), harvested and plated onto a 2-well Culture-Insert (ibidi, Martinsried, Germany) according to the manufacturer’s instructions. The Culture-Inserts were removed after 16 h, and cell migration was monitored over the indicated timepoints. For cell invasion assay, biocoat matrigel invasion chambers with 8-μm pores in 24-well plates (Corning, Discovery Labware, Inc., Bedford, MA, USA) were used. 24 h after treatment with CCNF-pcDNA3.1 (or pcDNA3.1 or PBS control), BRCA cells were plated onto the chambers. The cells were detached with 0.05 % Trypsin-EDTA, resuspended in conditioned medium (10 % FBS) and added to the upper chambers, according to the manufacturer’s instructions. After 24 h of incubation at 37 °C, the cells on the upper

chamber were completely removed by wiping with a cotton swab, and then the filter was fixed with 100 % methanol and stained with crystal violet solution (0.5 % (w/v) crystal violet in 25 % (v/v, methanol). Cells that had migrated from the upper to the lower side of the filter were imaged and counted with a light microscope (5 fields/filter).

## References


[1] ChangSC, HsuW, SuECY, HungCS, DingJL. Human fbxl8 is a novel e3 ligase which promotes brca metastasis by stimulating pro-tumorigenic cytokines and inhibiting tumor suppressors. Cancers (Basel) 2020;12:1–21. https://doi.org/10.3390/cancers12082210.
