# Supplementary material for: A Novel Signature of CCNF-Associated E3 Ligases Collaborate and Counter Each Other in Breast Cancer
Source: Cancers (Basel). 2021 Jun 8;13(12):2873. doi: 10.3390/cancers13122873 (PMC8228695; doi:10.3390/cancers13122873)
Supplement: Supplementary file 1 [file cancers-13-02873-s001.zip › suppl+ WB/Supplementary Tables and Figures.pdf]

Supplementary Tables S1, S2 & Supplementary Figures S1-S5

Supplementary Table S1. Clinicopathological parameters in paired primary breast carcinoma tissues used for global RNA-seq analysis.

Patient	1	2	3	4	5
Diagnosis	IDC	IDC	IDC	ILC	IDC
Histologic type	IDC	IDC	IDC	ILC	IDC
Histologic grade	2	2	3	2	3
pT	T1c	T2	T2	T3	T3
pN	N0	N0	N0	N0	N3a
pM	NA	NA	NA	NA	NA
Stage	IA	IIA	IIA	IIB	IIIC
ER	+	+	-	+	+
PR	+	+	-	+	-
HER2	1+	0	2+	2+	2+
# Ki67	25%	10%	25%	15%	30%
Age (year)	69	38	59	69	59
Gender (M/F)	F	F	F	F	F

Ki67 is an intracellular marker for cell proliferation. Generally, in breast cancer, the expression of Ki67 of >14% is taken as “positive” and the patients are advised to go for chemotherapy.

Supplementary Table S2. Clinicopathological parameters in primary BRCA tissues used for IHC analysis.

A

Patient	38	39	40	41	42	43	44	45	46
Diagnosis	IDC	IDC	IDC	IDC	IDC	IDC	IDC	IDC	IDC
Histologic type	IDC	IDC	IDC	IDC	IDC	IDC	IDC	IDC	IDC
Histologic grade	1	2	3	3	1	2	3	3	3
pT	T1c	T1b	T1c	T1c	T1c	T1c	T1c	T1c	T1c
pN	N0	N0	N0	N0	N0	N0	N0	N0	N1
pM	M0	M0	M0	M0	M0	M0	M0	M0	M0
Stage	IA	IA	IA	IA	IA	IA	IA	IA	IB
ER	+	-	+	+	+	+	+	-	-
PR	+	-	-	+	+	+	-	-	-
HER2	2+	0	2+	2+	2+	2+	3+	0	2+
Ki67	10%	25%	15%	10%	15%	50%	20%	70%	10%
Age (year)	59	30	46	63	60	71	51	51	58
Gender (F/M)	F	F	F	F	F	F	F	F	F

B

Patient	47	48	49	50	51	52	53	54	55	56	57
Diagnosis	IDC	IDC	IDC	IDC	IDC	IDC	IDC	IDC	IDC	IDC	IDC
Histologic type	IDC	IDC	IDC	IDC	IDC	IDC	IDC	IDC	IDC	IDC	IDC
Histologic grade	2	3	3	3	2	1	2	3	2	2	3
pT	T2	T2	T1c	T2	T2	T1c	T2	T2	T2	T2	T2
pN	N0	N0	N1	N0	N0	N1	N1	N1	N1	N1	N1
pM	M0	M0	M0	M0	M0	M0	M0	M0	M0	M0	M0
Stage	IIA	IIA	IIA	IIA	IIA	IIA	IIB	IIB	IIB	IIB	IIB
ER	-	+	-	-	-	+	+	+	+	+	-
PR	-	+	-	-	-	-	+	+	+	+	-
HER2	1+	1+	3+	1+	0	1+	2+	0	1+	0	3+
Ki67	25%	40%	40%	50%	75%	5%	10%	40%	5%	5%	50%
Age (year)	60	39	46	56	51	48	66	80	51	51	56
Gender (F/M)	F	F	F	F	F	F	F	F	F	F	F

C

Patient	58	59	60	61	62	63	64	65	66	67
Diagnosis	IDC	IDC	IDC	IDC	IDC	IDC	IDC	IDC	IDC	IDC
Histologic type	IDC	IDC	IDC	IDC	IDC	IDC	IDC	IDC	IDC	IDC
Histologic grade	2	2	3	3	3	2	2	2	3	3
pT	T3	T2	T2	T3	T2	T2	T2	T2	T3	T3
pN	N2	N2	N1	N2	N2	N2	N2	N2	N2	N2
pM	M0	M0	M0	M0	M0	M0	M0	M0	M0	M0
Stage	IIIA	IIIA	IIIA	IIIA	IIIA	IIIA	IIIA	IIIA	IIIA	IIIA
ER	+	+	-	-	+	+	-	+	+	+
PR	+	+	-	-	+	+	-	+	+	+
HER2	1+	3+	1+	0	2+	1+	0	0	2+	0
Ki67	50%	65%	40%	60%	8%	20%	70%	30%	50%	30%
Age (year)	42	57	74	50	72	38	72	51	34	70
Gender (F/M)	F	F	F	F	F	F	F	F	F	F

Supplementary Figure S1. The efficacy of siRNA knockdown of FBXO43 in MCF7 and MDA-MB231 cells.

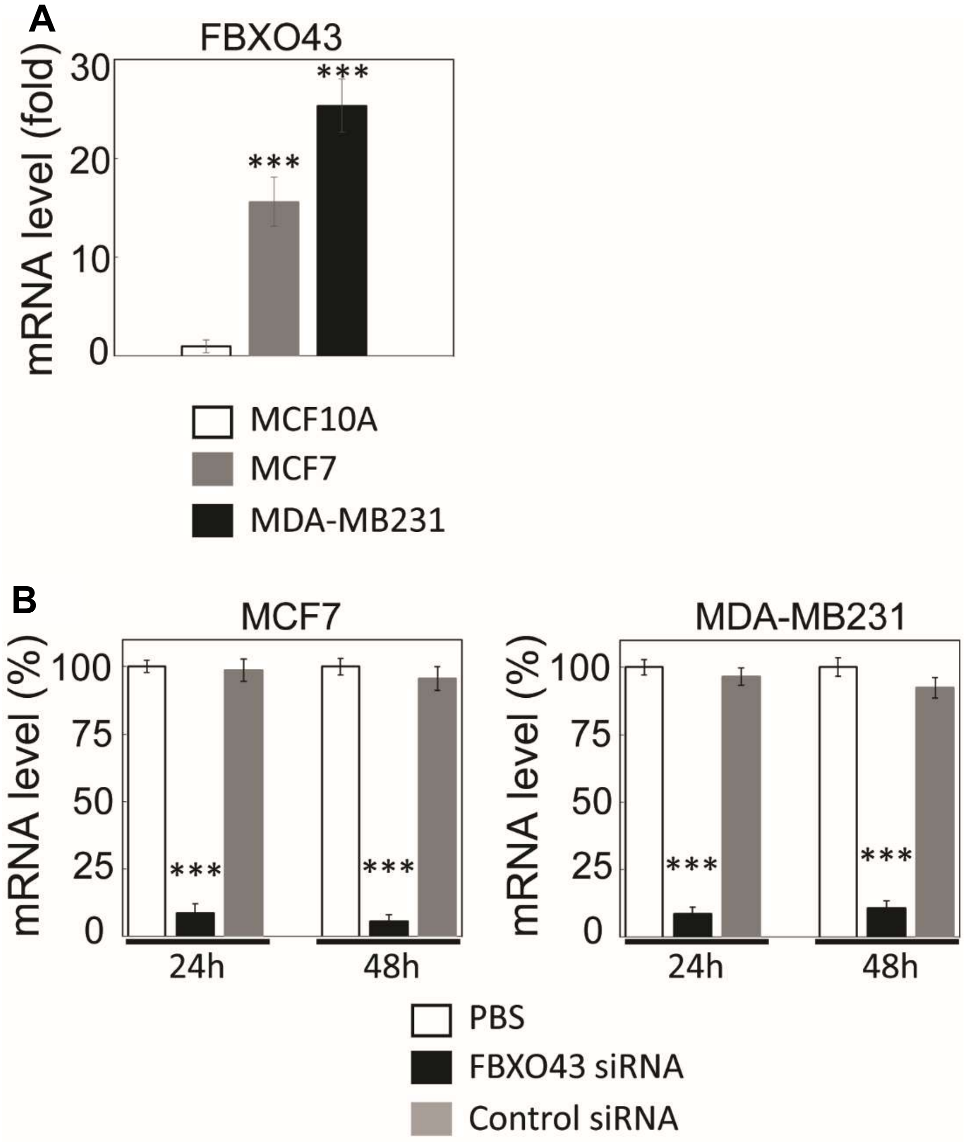

Supplementary Figure S2. The efficacy of siRNA knockdown of FBXO15 in MCF7 and MDA-MB231 cells.

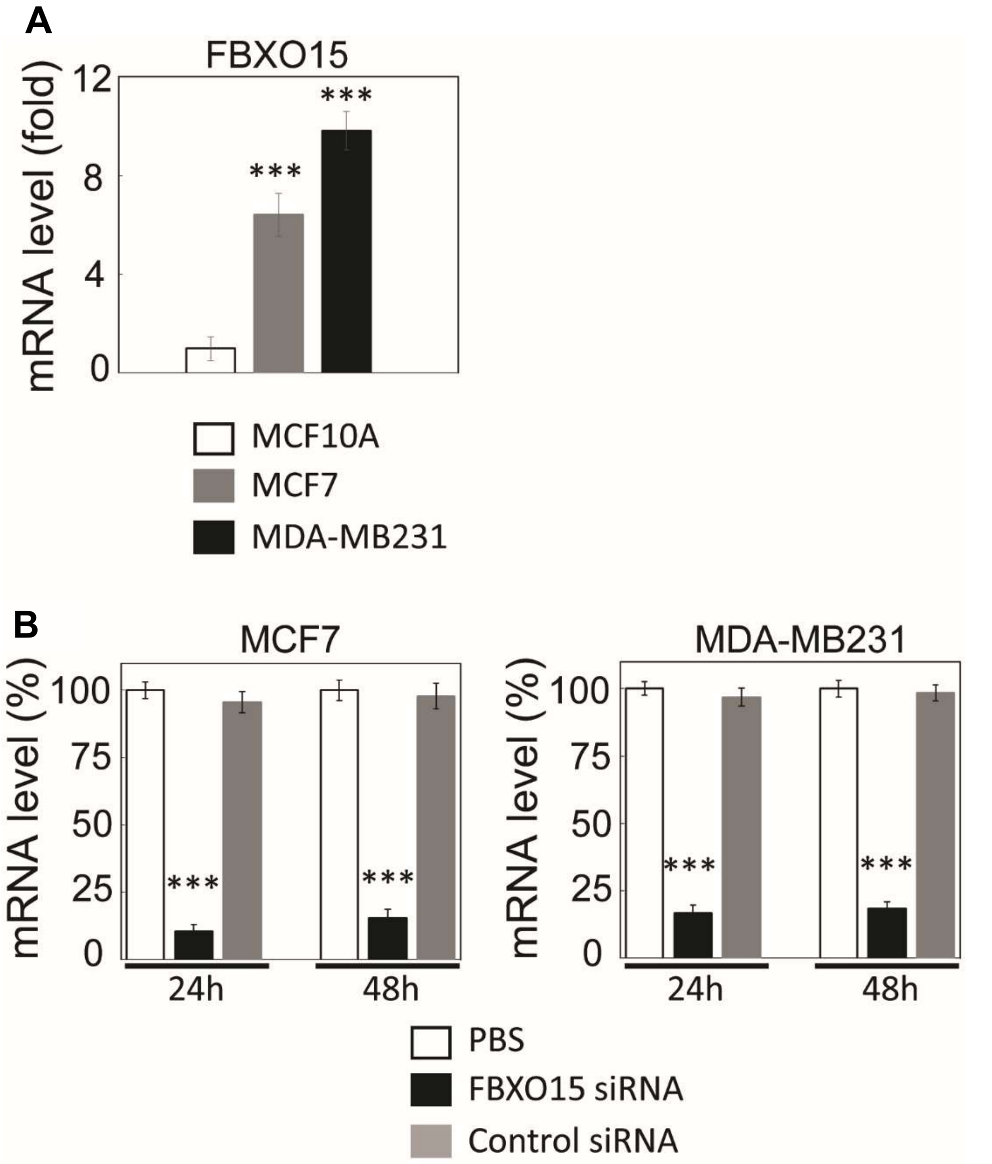

Supplementary Figure S3. The efficacy of siRNA knockdown and pcDNA3.1-overexpression of CCNF in MCF7 and MDA-MB231 cells.

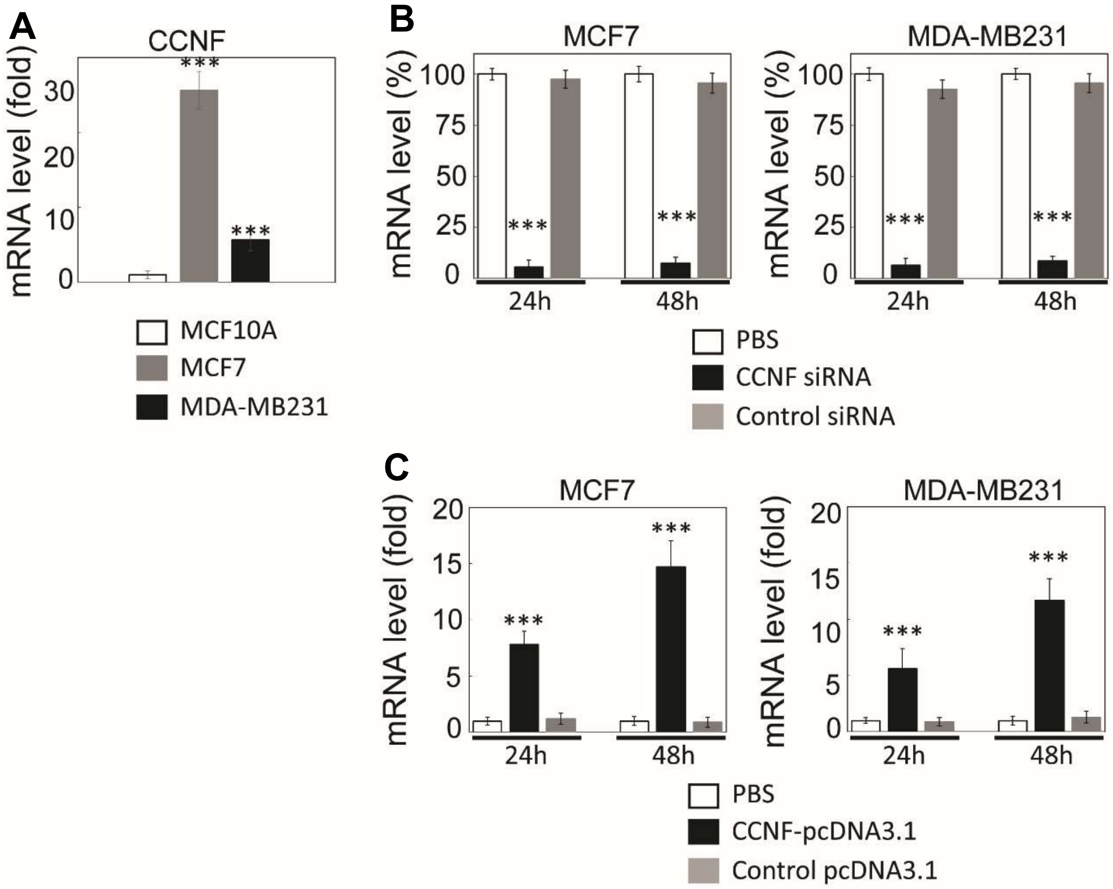

Supplementary Figure S4. FACS analysis of apoptosis after pcDNA3.1 transfection treatments of breast cancer cells.

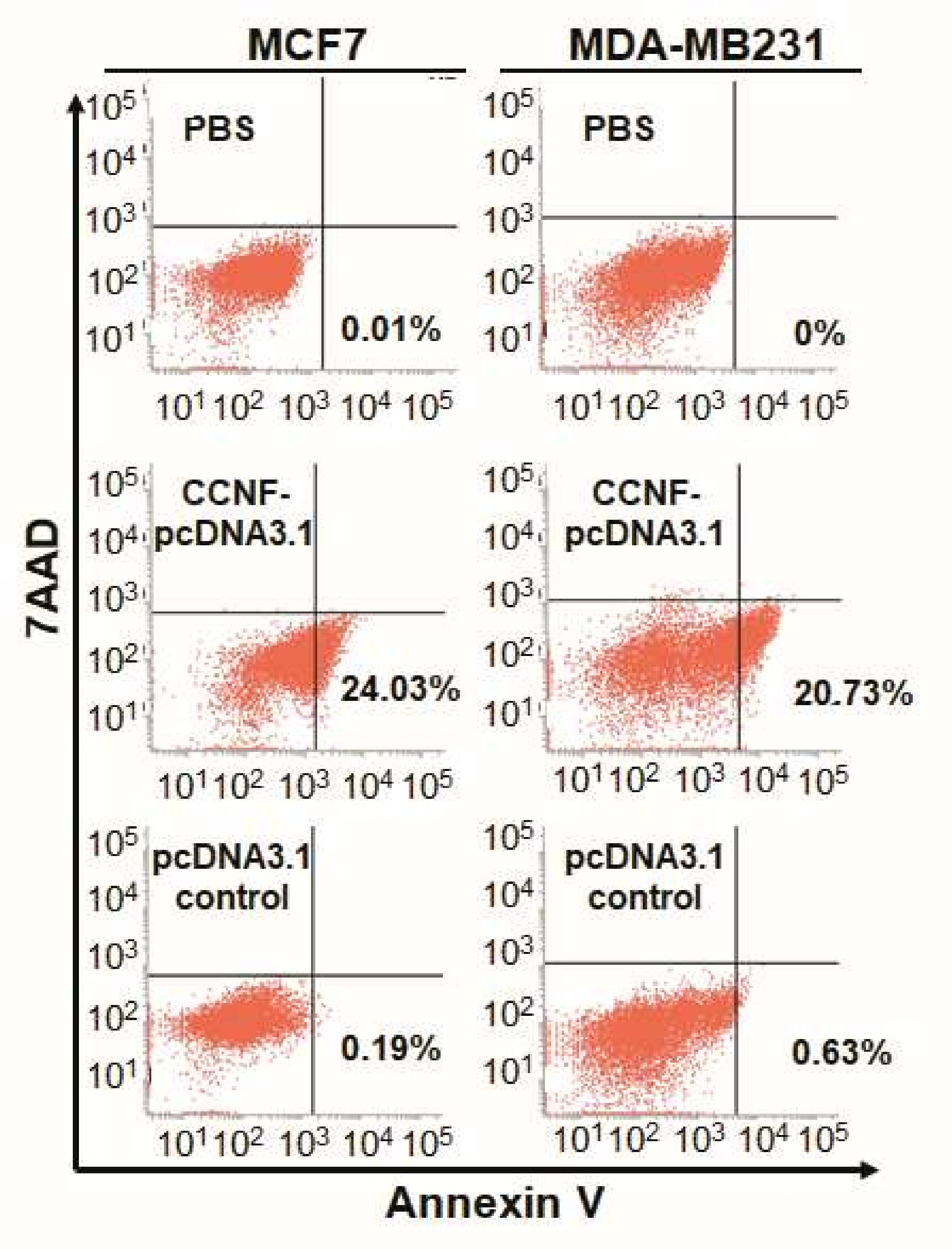

Supplementary Figure S5. Representative microscopy images of breast cancer cell invasion.

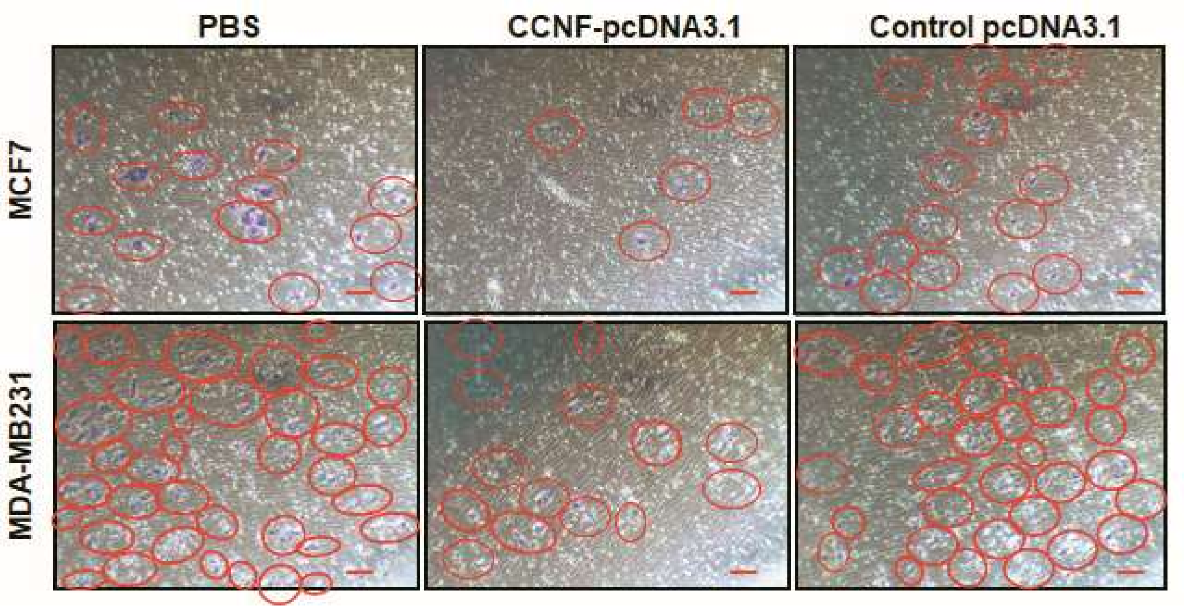

=

Supplementary Table & Figure legends

Supplementary Table S1. Clinicopathological parameters in paired primary breast carcinoma tissues used for global RNA-seq analysis. Clinicopathological information provided from TMUH, includes diagnosis, histological type, histological grade, pTNM Pathological Classification, stage, ER, PR, HER2, Ki67, age and gender in respective tissue. Paired primary tissues from breast carcinoma (n=5) and corresponding normal breast tissues (n=5) were examined. F, female; IDC, Invasive ductal carcinoma; ILC, Invasive lobular carcinoma; ER, estrogen receptor; PR, progesterone receptor; pN, pathologic lymph node status; pT, Primary Tumour; pM, Distant Metastasis.

Supplementary Table S2. Clinicopathological parameters in primary BRCA tissues used for IHC analysis. To affirm the transcriptional data, we retrospectively studied primary breast tissues based on the IHC analysis. Clinicopathological information provided from TMUH, include diagnosis, histological type, histological grade, pTNM Pathological Classification, stage, ER, PR, HER2, Ki67, age and gender in respective tissues. A total 60 primary tissues were examined, constituting breast carcinoma (n=30) and paired normal breast tissues (n=30). Samples were categorized into three groups based on cancer staging. (A): IA/IB as initial stage (n=18, including n=9 carcinoma tissues and n=9 normal breast tissues); (B): IIA/IIB as middle stage (n=22, including n=11 carcinoma tissues and n=11 normal breast tissues) and (C): IIIA as late stage (n=20, including n=10 carcinoma tissues and n=10 normal breast tissues). F, female; IMC, Invasive mammary carcinoma; IDC, Invasive ductal carcinoma; ILC, Invasive lobular carcinoma; MC, Mucinous carcinoma; MMC, Mixed mucinous carcinoma; DCIS, Ductal carcinoma in situ; fc, fold change; ER, estrogen receptor; PR, progesterone receptor; pN, pathologic lymph node status; pT, Primary Tumour; pM, Distant Metastasis.

Supplementary Figure S1. The efficacy of siRNA knockdown of FBXO43 in MCF7 and MDA-MB231 cells. (**A**) The endogenous levels of FBXO43 were examined in two BRCA cell lines (MCF7 and MDA-MB231) and a control non-cancer cell line (MCF10A). (**B**) To examine FBXO43 RNAi efficacy in BRCA cells, FBXO43-sequence-specific siRNAs were transfected into the cells for 24h and 48h. Then a quantitative real-time PCR for FBXO43 was processed in MCF7 and MDA-MB231 cells. The mRNA expression levels from different treatments were compared with PBS-treatment condition. All expression values were normalized against GAPDH as an endogenous control. ***, $p<0.001$.

Supplementary Figure S2. The efficacy of siRNA knockdown of FBXO15 in MCF7 and MDA-MB231 cells. (**A**) The endogenous levels of FBXO15 were examined in two BRCA cell lines (MCF7 and MDA-MB231) and a control non-cancer cell line (MCF10A). (**B**) To examine

FBXO15 RNAi efficacy in BRCA cells, FBXO15-sequence-specific siRNAs were transfected into the cells for 24h and 48h. Then a quantitative real-time PCR for FBXO15 was processed in MCF7 and MDA-MB231 cells. The mRNA expression levels from different treatments were compared with PBS-treatment condition. All expression values were normalized against GAPDH as an endogenous control. ***, p<0.001.

Supplementary Figure S3. The efficacy of siRNA knockdown and pcDNA3.1-overexpression of CCNF in MCF7 and MDA-MB231 cells. (**A**) The endogenous levels of CCNF were examined in two BRCA cell lines (MCF7 and MDA-MB231) and a control non-cancer cell line (MCF10A). (**B**) To examine CCNF RNAi efficacy in BRCA cells, CCNF-sequence-specific siRNAs were transfected into the cells for 24h and 48h. Then a quantitative real-time PCR for CCNF was processed in MCF7 and MDA-MB231 cells. The mRNA expression levels from different treatments were compared with PBS-treatment condition. (**C**) To examine CCNF overexpression efficacy in BRCA cells, CCNF-pcDNA3.1 (or empty pcDNA3.1 vector as a control) were transfected into the cells for 24h and 48h. Then a quantitative real-time PCR for CCNF was processed in MCF7 and MDA-MB231 cells. The mRNA expression levels from different treatments were compared with PBS-treatment condition. All expression values were normalized against GAPDH as an endogenous control. ***, p<0.001.

Supplementary Figure S4. FACS analysis of apoptosis after pcDNA3.1 transfection treatments of breast cancer cells. Representative histograms of cell apoptosis assay (Annexin V and 7AAD double staining), including controls (PBS and pcDNA3.1 control). Two breast cancer cell lines are tested, including MCF7 and MDA-MB231. Early apoptotic cells are indicated by Annexin V^+/$7AAD^-$ (shown as %).

Supplementary Figure S5. Representative microscopy images of breast cancer cell invasion. 24 h after treatment of breast cancer cells with CCNF-pcDNA3.1, control pcDNA3.1 or PBS control, matrigel invasion assay was performed. Expression of CCNF efficiently suppressed breast cancer invasion. Two breast cancer cell lines are tested, including MCF7, and MDA-MB231. After another 24 h incubation, cells that had migrated from the upper to the lower side of the filter were imaged (red circle highlights) and counted with a light microscope (5 fields/filter). Scale bar is 100 μm, shown as the red color line (—).
